# Parental and Peer Support are Associated with Physical Activity in Adolescents: Evidence from 74 Countries

**DOI:** 10.3390/ijerph17124435

**Published:** 2020-06-20

**Authors:** Shanchita R. Khan, Riaz Uddin, Sandra Mandic, Asaduzzaman Khan

**Affiliations:** 1 School of Public Health and Social Work, Queensland University of Technology (QUT), Kelvin Grove QLD 4059, Australia; shanchita.khan@qut.edu.au; 2 Institute for Physical Activity and Nutrition (IPAN), School of Exercise and Nutrition Sciences, Deakin University, Geelong, VIC 3220, Australia; r.uddin@deakin.edu.au; 3 School of Health and Rehabilitation Sciences, The University of Queensland, Brisbane, QLD 4072, Australia; 4 Active Healthy Kids Bangladesh (AHKBD), Dhaka, Bangladesh; 5 Active Living Laboratory, School of Physical Education, Sport and Exercise Sciences, University of Otago, P.O. Box 56, Dunedin 9054, New Zealand; sandra.mandic@otago.ac.nz; 6 Centre for Sustainability, University of Otago, Dunedin 9054, New Zealand

**Keywords:** parent support, peer support, physical activity, adolescent, children, global health, school health

## Abstract

Although parental and peer support can influence adolescents’ physical activity (PA), these associations have not been fully examined through a global assessment. This study examined the associations of parental and peer support with PA among adolescents from 74 countries. The Global School-based Student Health Survey data from 250,317 adolescents aged 11–17 years (48.8% girls), collected between 2007 and 2016, were analysed. Adolescents were asked how many days/week they were physically active and about their parental and peer support. Meta-analysis showed that adolescents who had high parental or peer support had higher odds of attaining sufficient PA (odds ratio (OR): 1.40, 95% confidence interval (CI): 1.34–1.46; OR: 1.57, 95% CI: 1.49–1.65, respectively). Pooled estimates of association were significant across all World Health Organization (WHO) regions and country-income categories with the highest estimate from the low-income countries. The Western Pacific region showed the highest association between parental support and adolescents’ PA (OR: 1.49, 95% CI: 1.41–1.59), while South-East Asia exhibited the highest association between peer support and adolescents’ PA (OR: 1.80, 95% CI: 1.59–2.04). Country-level estimates of associations are presented. Future studies should use robust assessment of PA and PA-specific parental and peer support with emphasis on qualitative investigation to understand the complexity of the relationships.

## 1. Introduction

Physical activity (PA) is essential for the well-being of adolescents and is associated with physical fitness, bone health, and cardio-metabolic biomarkers such as cholesterol, blood pressure, insulin and glucose levels [1]. Nevertheless, the vast majority of adolescents are not sufficiently active to achieve the health benefits. Globally, more than 80% of adolescents do not meet the recommended guidelines of the World Health Organization (WHO) to accumulate ≥60 min of moderate to vigorous PA (MVPA) per day [2,3]. Insufficient PA also can co-occur with other lifestyle risk factors such as sedentary behaviours, tobacco smoking, alcohol abuse, and poor dietary habits among adolescents [4]. Physically inactive lifestyles have become a major public health challenge for adolescents worldwide [3], and is one of the factors contributing to an epidemic of overweight and obesity among children and adolescents in both developed and developing countries [5]. 

Parents have a strong influence on the lives of their children and can positively affect adolescents’ PA levels through encouragement, involvement and facilitation [6]. Of all the individual behaviours constituting parental support, verbal and non-verbal encouragement has the greatest effect on children’s PA [7] and has been positively associated with the intensity of PA in adolescents [8]. Parental support through providing transportation and equipment is also associated with increased PA in children and adolescents [9,10]. In addition, when parents co-participate in PA, their children and adolescents are more likely to meet the PA guidelines [9]. Studies examining gender differences in parental support have yielded mixed results. While a number of studies have reported no significant differences in the influence of fathers or mothers on their children’s PA [11,12], there is evidence to suggest that parents may provide greater support for PA to boys compared to girls [13,14]. However, little is known about the interactions between parental support and environmental factors (e.g., safety, access to PA facilities) or cultural factors (e.g., ethnicity, social class, gender expectation) which may influence children’s PA.

While previous studies showed some relationships between parental support and adolescents’ PA, the role of peer support on adolescents’ PA is not well understood yet. A limited number of studies have reported a positive association between peer influence and adolescents’ PA [8,15,16]. Studies from the USA reported that adolescents who received peer support showed higher odds of engaging in the recommended levels of daily PA and lower odds of engaging in two or more hours of screen-time per day [8,10,14]. A 5-year longitudinal study in Scottish adolescents found that while parental support decreased with the increase of adolescents’ age, peer support became more important in determining PA levels [14]. However, whether the level of adolescents’ autonomy in making lifestyle decisions has any influence on their PA is yet to be understood.

Although there is evidence to suggest associations between parental and peer support and adolescents’ PA levels, such evidence is based on studies conducted in single countries. Only a handful of multi-country studies conducted on this topic to date [17,18] reported positive associations between parental involvement and their children’s PA; however, these studies were based on Asian and Pacific Island countries, and did not explore the role of peer support on adolescents’ PA. More extensive multi-country examinations of associations of parental and peer support with adolescents’ PA with wider cultural diversity are particularly important as the approaches, mechanisms and levels of such support may vary across cultures, regions or countries. While Western countries are often characterised by a focus on an individual (i.e., individualistic culture), most of the non-Western developing countries share collectivistic cultures where individuals view themselves as interdependent within their groups [19]. Thus, adolescents from collectivistic cultures may find themselves more prone to peer influence than their counterparts from individualistic cultures [20]. The current understanding of the relationship is lacking the diversity of adolescents’ socio-economic status and cultural differences in a global scale. More inclusive and multi-country studies are, therefore, essential to obtain a comprehensive global perspective on the relationship between parental and peer support and adolescents’ PA levels. Therefore, using nationally representative data from 74 countries across different regions, this study investigated the associations of parental and peer support with PA levels of adolescents around the globe.

## 2. Materials and Methods

This study was based on publicly available data from countries that participated in the population-based survey of school-going children and adolescents around the world, the Global School-based Student Health Survey (GSHS) [21]. GSHS is a WHO and US Centers for Disease Control and Prevention (CDC) initiative, in collaboration with the United Nations Children’s Fund (UNICEF), the United Nations Educational, Scientific and Cultural Organization (UNESCO), and the Joint United Nations Program on HIV/AIDS (UNAIDS). As a global health surveillance system for school-going students, the GSHS provides data on different aspects of adolescent behaviours (e.g., physical activity) and protective factors (e.g., parental and peer support) with an aim to help countries develop suitable school and adolescent health programmes and policies, and to facilitate comparison of these behaviours and factors across countries [21,22]. The GSHS uses the same standardised sampling technique and study methodology in all participating countries. Survey participants completed a standardised self-administered anonymous questionnaire, which included, but was not limited to, questions on demographics (e.g., age, gender, food insecurity), PA, parental and peer support. The GSHS was administered in a classroom, and the survey completion took approximately 40–45 min. Students’ responses to each of the questions were recorded on a computer scan-able answer script. Using an automated optic character recognition system, data entry was completed automatically at the US CDC [21,22].

From all publicly available GSHS data between 2007 and 2016, we selected the datasets that included the key variables pertaining to this analysis. For countries with more than one GSHS dataset, we used the most recent one available. Three countries (Niue, Tokelau, and Montserrat) were excluded from the analysis due to their small sample size (n ≤ 210). The final analytical sample consists of 250,317 adolescents aged 11–17 years from 74 countries, including seven low-income, 25 lower-middle-income, 22 upper-middle-income, and 19 high-income countries, based on the World Bank classification at the time of the survey. Information on Cook Island’s income classification was not available. Of the participating countries, seven were from Africa, 22 from the Americas, 19 from the Eastern Mediterranean, one from Europe, eight from South-East Asia, and 17 from the Western Pacific region. GSHS used a two-stage cluster-sampling design where at the first stage, schools were drawn with probability proportional to their enrolment size and at the second stage, a random selection procedure was used to select classes from the schools. All students in those selected classes were eligible to participate [21,22]. All countries included in this current study, except Ecuador, provided nationally representative samples.

The GSHS received ethics approval from the Ministry of Education or a relevant Institutional Ethics Review Committee, or both in each of the participating countries. Only those adolescents and their parents who provided written or verbal consent participated. As the current study used retrospective, de-identified, publicly available data, ethics approval was not required for this secondary analysis. Detailed methods of the GSHS have been described on both the US CDC and the WHO websites [21,22].

### 2.1. Outcome Measure—Physical Activity

PA was assessed with one item: ‘During the past 7 days, on how many days were you physically active for a total of at least 60 min per day?’ The response options were 0 to 7 days. Consistent with the WHO recommendations [2], we defined participants as ‘sufficiently active’ who reported engaging in ≥ 60 min/day of PA on 7 days of the week.

### 2.2. Study Factors—Parental Involvement and Peer Support

Parental support was assessed with one item: “During the past 30 days, how often did your parents or guardians understand your problems and worries?”, while peer support was assessed with another item: “During the past 30 days how often were most of the students in your school kind and helpful?”. Possible response options to each of these items were ‘never’, ‘rarely’, ‘sometimes’, ‘most of the time’, and ‘always’. For the regression modelling purpose, the response options were dichotomised: ‘sometimes/rarely/never’ as ‘low levels’ and ‘always/most of the times’ as ‘high levels’ [17].

### 2.3. Covariates

Age and gender of the participants were included in the survey. The participants were also asked: “During the past 30 days, how often did you go hungry because there was not enough food in your home?” with response options being ‘never’, ‘rarely’, ‘sometimes’, ‘most of the time’, and ‘always’. This food insecurity variable was used as a proxy of socioeconomic status [23,24] as the GSHS did not include any direct measure of socioeconomic status. Trained survey staff measured participants’ height and weight [23]. Body mass index (BMI) was categorised as underweight (BMI <−2 Standard Deviation [SD]), overweight (BMI > +1SD), and obese (BMI > +2SD), relative to median BMI, by age and gender based on the WHO Child Growth Standards [21].

### 2.4. Statistical Analyses

Country-specific estimates of prevalence of PA, and parental and peer support were computed by taking into account the weighting factor that was applied to each student record to adjust for non-response and the varying probability of selection. Within the GSHS protocol, weighting accounted for the probability of selection of schools and classrooms, non-responding schools and students, and distribution of the population by gender and grade. In examining the association of parental and peer support with PA, a set of covariates was considered including age, gender, BMI, and food insecurity. Possible interactions between gender and parental support, and gender and peer support were tested to see whether gender based analysis was warranted. As the interactions for the overall sample were not statistically significant, the regression modelling was implemented on the overall sample without any stratification by gender, as done elsewhere [25]. A linear trend test was conducted across ordered groups of parental and peer support and adolescents’ PA.

Given the dichotomous nature of the outcome (PA), binary logistic regression analysis with robust standard errors was used to examine the associations at the country level, by taking into account the sampling weight. This GSHS weighting factor was applied in an identical way to estimate the associations in each of the participating countries. To generate pooled estimates of the association, random effects meta-analysis with the DerSimonian and Laird inverse-variance method were used. Pooled estimates were also derived by country income category, using the World Bank country income classification, collected at the time of the survey for the respective countries, and by WHO regions. metan, a Stata meta-analysis routine, was used to estimate the pooled odds ratios with 95% CI for the estimates. All adjusted estimates of the association parameters are presented in the form of odds ratio (OR) and 95% confidence interval (CI). All statistical analyses were performed using Stata 14.0 SE.

## 3. Results

The mean age of participating adolescents (n = 250,317) was 14.3 (SD = 1.43; range 11–17) years and 48.8% were girls. The vast majority of the participants were from the lower-middle income countries (68.7%), while upper-middle income countries represented 14.7% and low income countries represented 13.5% of the participating adolescents. Over a third (38.2%) of participants were from South-East Asia, followed by 24.3% from Western Pacific and 22.0% from the Eastern Mediterranean region. About 6.1% of adolescents went hungry most of the time or always because there was not enough food in their home, and 44.0% went hungry occasionally. About 15% of adolescents were overweight or obese, while 9.3% were underweight.

**Figure 1:**
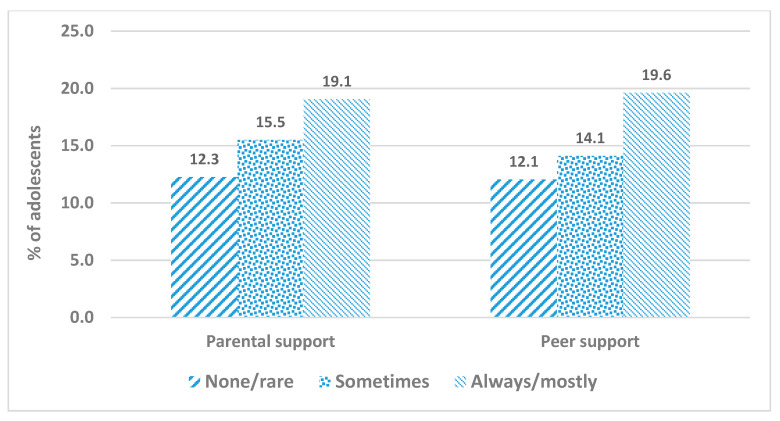
Prevalence of adolescents’ sufficient physical activity by levels of parental and peer support using the Global School-based Student Health Survey, 2007–2016.

The prevalence of sufficient PA among adolescents was 15.6% (95% CI: 15.3–15.9). More than one-third of adolescents reported high levels (‘always or most of the time’) of parental support (37.5%; 95% CI: 37.1–37.9), while high levels of peer support was reported by 43% of adolescents (95% CI: 42.8%; 42.4–43.3). Overall, compared to boys, slightly higher proportion of girls reported high levels of parental and peer support (38.6% vs. 36.6%, and 45.6% vs. 40.1%, respectively). High levels of parental and peer support were more prevalent among girls than boys in 69% and 89%, respectively, of the participating countries. The prevalence of sufficient PA among adolescents increased linearly with the increase of levels of parental support (p_trend_ < 0.001) and peer support (p_trend_ < 0.001) (Figure 1). About one-fifth of adolescents (~19%) with high levels of parental or peer support reported sufficient PA compared to 12% of their counterparts who reported low levels of parental or peer support.

Country-wise estimates of association of parental and peer support with sufficient levels of adolescents’ PA are shown in Table 1. The country-level analysis showed that 49 out of 74 countries (66%) demonstrated significant and positive association between high levels of parental support and adolescents’ sufficient PA. Twenty-eight countries (38%) showed that their adolescents had at least 50% higher odds (OR ≥ 1.50) of being sufficiently active when high levels of parental support were present. For example, Jordanian adolescents who had high levels of parental support had double the odds of reporting sufficient PA compared to their counterparts who reported low levels of parental support (OR: 2.05, 95% CI: 1.50–2.82). High levels of peer support was positively and significantly associated with adolescents’ sufficient PA in 57 out of 74 countries studied (77%). Forty-three countries (58%) demonstrated that their adolescents had at least 50% higher odds (OR ≥ 1.50) of being sufficiently active when high levels of peer support were present. For example, Bangladeshi adolescents with high levels of peer support had 2.5 times higher odds of reporting sufficient PA compared to their counterparts who reported low levels of peer support (OR: 2.46, 95% CI: 1.93–3.14).

Overall, there was no gender difference in the estimates of associations between parent and peer support and adolescents’ PA, as supported by the insignificant parent-support*gender and peer-support*gender interactions. For parental support, the overall association estimate (OR) was 1.35 (95% CI: 1.26–1.44) for boys and 1.43 (95% CI: 1.33–1.53) for girls. For peer support, the overall association estimate (OR) was 1.53 (95% CI: 1.43–1.63) for boys and 1.60 (95% CI: 1.49–1.72) for girls.

**Figure 2:**
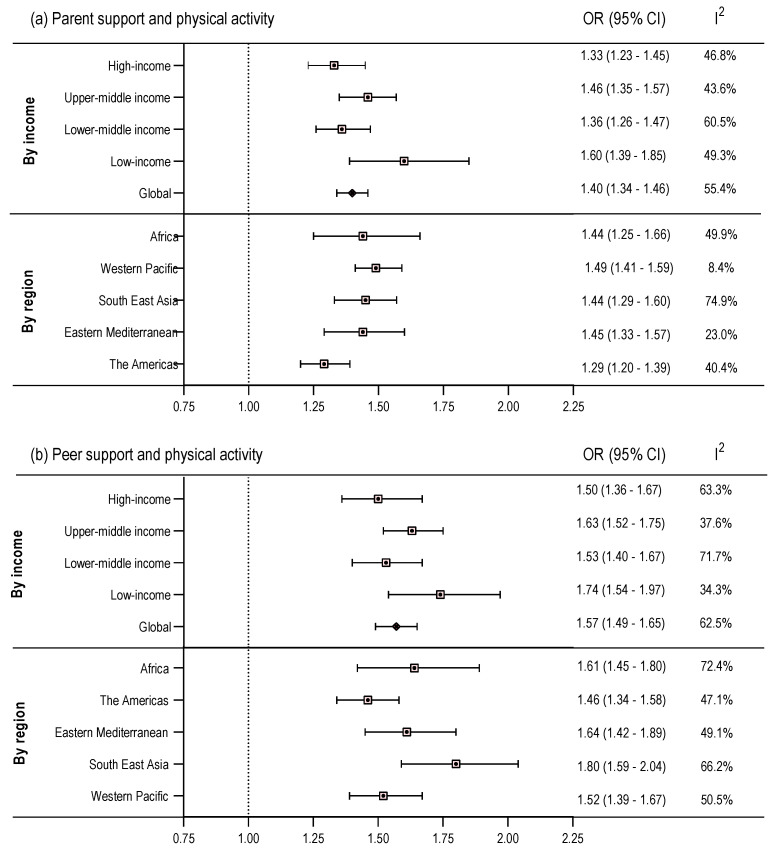
Pooled odds ratios (ORs) of association of high levels of (**a**) parental support and (**b**) peer support with sufficient physical activity among adolescents aged 11–17 years, by World Bank country income classification, and WHO region, Global School-based Student Health Survey, 2007–2016. [I-squared represents variation in effect size (OR) attributable to heterogeneity].

The pooled estimates of associations from random effect meta-analyses are presented in Figure 2. Adolescents with high levels of parental support had 40% higher odds of reporting sufficient PA compared to their counterparts with low levels of parental support (OR: 1.40, 95% CI: 1.34–1.46). Similarly, odds of reporting sufficient PA was 57% higher among adolescents who reported high levels of peer support compared to those who reported low levels of peer support (OR: 1.57, 95% CI: 1.49–1.65). Analyses by country income showed significant associations in all country income groups, with no significant differences across the groups. Low-income countries showed the highest pooled estimate of association for both parental (OR: 1.60, 95% CI: 1.39–1.85) and peer support (OR: 1.74, 95% CI: 1.54–1.97). High-income countries showed the lowest pooled estimate of association for both parental (OR: 1.33, 95% CI: 1.23–1.45) and peer support (OR: 1.50, 95% CI: 1.36–1.67). In all WHO regions, high levels of parental and peer support were significantly and positively associated with reporting sufficient PA. The differences in association estimates were marginally significant between the Americas and Western Pacific region for parental support and adolescents’ PA, and between the Americas and South-East Asia region for peer support and adolescents’ PA.

## 4. Discussion

To the authors’ knowledge, this is the largest multi-country study examining the associations of parental and peer support with PA of adolescents representing 74 countries across all WHO regions and with various country income levels. After adjusting for age, gender, BMI and food insecurity (as a proxy of socio-economic status), the modelling showed that adolescents with high levels of parental and/or peer support had significantly higher odds of attaining the daily recommended levels of PA. The pooled estimates of association were significant across all WHO regions and country-income groups. The highest pooled estimates of association for both parental and peer support were found in the low-income countries, while South-East Asia region exhibited the highest estimates of association between peer support and adolescents’ PA.

In the present study, the prevalence of sufficient PA among adolescents increased linearly with the increase of levels of parental support, as demonstrated by the significant p_trend_ value. The highest pooled associations between parental support and adolescents’ PA were found in the Western Pacific region and in low-income countries. Parental support has been consistently reported as a positive correlate of adolescents’ PA in previous studies [6,8,9,10]. Parents and children may engage in PA together; parents may act as a role model by setting examples for their children, and facilitate home environments that foster PA and discourage sedentary lifestyle (e.g., watching TV viewing) [6]. Parents can also facilitate or discourage activity behaviours such as active transport to/from school, which represent a daily opportunity for adolescents’ PA. For example, parental perceptions of different modes of transport to school, including walking and cycling, are associated with adolescents’ mode choice for school travel [26,27]. A recent study from New Zealand shows that two thirds of parents expected to participate in decision-making regarding walking and/or cycling to school for their adolescents [28], further reinforcing that parents play a critical role in decision-making regarding behaviours that may influence PA levels of their adolescents. The results of the present study show that parental support is critical for achieving sufficient PA in adolescents across diverse cultures and WHO regions and particularly important in low-income countries.

During adolescence, children spend considerable time with their peers and friends, and over time, they become more independent of their parents [29]. During this time, peers can provide motivation and help in making for healthy lifestyle choices such as influencing each other’s PA participation [30]. In the present study, adolescents who had high levels of peer support had higher odds of attaining sufficient PA, and these findings of significant associations were consistent across all WHO regions and country-income categories. The highest pooled associations between peer support and adolescents’ PA were found in the South-East Asia region and in low-income countries. A cross-cultural comparison study reported competition and improving skills to be the main motivating factors for US adolescents’ engagement in PA, while Chinese adolescents mainly engaged in PA for social affiliation and wellness [31]. Another study examining peer support in more detail in Chinese adolescents found that peer support was not directly associated with PA but rather peer support was indirectly associated with PA through either self-efficacy or enjoyment of PA [32]. The present study also found that adolescents received more support from peers than parents, which is consistent with the previous studies [8,14]. Therefore, in addition to parental support, future initiatives to increase PA among adolescents should also aim to encourage peer support for PA, particularly in South-East Asian countries.

In our study, adolescents’ age was not associated with the levels of PA when considered in the analyses as an adjustment factor. There was no gender difference in the association between parental and peer support, and PA with girls reporting only slightly higher levels, although not statistically significant, of both parental and peer support than boys. In contrast, previous studies conducted in the US, Germany and Scotland reported that compared to boys, girls might receive less peer support for PA, and they might be engaging in more ‘non-physically active’ activities with their peers [8,10,14]. However, studies conducted in China reported that parents do not differentiate between sons and daughters when providing support [33,34], suggesting that some cross-cultural differences may exist. In certain conservative cultures, however, parental, especially mother’s education has been found to have an influence on girls’ PA [35]. A study conducted in Denmark found that while adolescent girls received significantly higher level of encouragement from their mothers, a higher proportion of boys than girls reported fathers watched them when they were physically active [12]. This suggests that there may be different dimensions of parental support in adolescents’ PA, which deserve further investigation particularly in a diverse cultural context.

Strengths of this study include nationally representative samples of school-going adolescents from 74 countries with diverse socio-cultural and economic context. The GSHS used the same standardised methods such as the type of sample (e.g., school-based), data collection procedures, and a standardised questionnaire with the same survey items to assess the variables of interest, which facilitated valid assessments of cross-country or regional differences. The statistical modelling of association estimates were adjusted for several potential covariates. The results presented were obtained by using weighted analyses, where the GSHS weighting accounted for the distribution of the population by gender and age.

While our study has a number of novel insights, the study findings should be interpreted with several limitations in mind. The GSHS data include only adolescents in schools and, therefore, information from adolescents who were unable to attend school or who had dropped out was not available, which compromises the generalisability of the findings. The degree of complexity in the relationships assessed might be influenced by cultural and/or gendered expectations of sport and recreation or availability of sport facilities, which were not captured in the GSHS. Sample sizes considered in the analyses were disproportionate across the participating counties with a quarter of the data coming from three countries. Parental and peer support were assessed using one item each that were referring to support in general and not specific to support for PA, and as such the support assessed may not fully encapsulate the constructs of parental or peer support under study. Data for this study are self-reported and, therefore, vulnerable to social desirability and recall bias. In addition, other factors (e.g., parent–child relationship, self-esteem) might have contributed to the self-reported perception of support of the survey participants; however, the GSHS did not collect information on these variables. Although GSHS data were collected in countries across all WHO regions, GSHS data were not available for most European countries and several other high-income countries including Australia, New Zealand, Canada and the United States. This study is limited by the cross-sectional design and, as such, causality cannot be established.

## 5. Conclusions

This study offers global evidence of strong and positive associations between high levels of parental and peer support and sufficient PA of adolescents across the globe. These findings add to the growing evidence of the cross-sectional link between parental and peer support and PA levels of adolescents. Prospective studies with robust assessment of PA and PA-specific parental and peer support that adolescents receive are required to further understand the associations. This study also underscores the need for qualitative explorations of the choices young people make about their engagement in PA.

## Figures and Tables

**Table 1:** Country-level adjusted odds ratios (ORs) of parental and peer support with sufficient levels of physical activity among adolescents aged 11–17 years, Global School-based Student Health Survey, 2007–2016.

Country	Analytical Sample Size	High Parental Support and Sufficient Physical Activity	High Peer Support and Sufficient Physical Activity
		OR ^1^ (95% Confidence Interval (CI))	OR ^1^ (95% CI)
Afghanistan	1855	1.59 (1.09–2.34)	2.01 (1.32–3.06)
Antigua and Barbuda	1097	1.25 (0.91–1.73)	1.51 (1.04–2.17)
Argentina	25,667	1.29 (1.11–1.49)	1.05 (0.91–1.22)
Bahamas	1228	1.34 (0.91–1.97)	1.96 (1.31–2.92)
Bahrain	6896	1.19 (1.03–1.37)	1.48 (1.27–1.73)
Bangladesh	2698	1.34 (1.06–1.69)	2.46 (1.93–3.14)
Barbados	1490	1.23 (0.89–1.71)	1.38 (0.96–1.99)
Belize	1949	1.13 (0.90–1.43)	1.35 (1.07–1.70)
Benin	1552	1.12 (0.86–1.46)	1.86 (1.40–2.48)
Bolivia	3357	1.66 (1.32–2.09)	1.63 (1.30–2.04)
British Virgin Island	1576	1.33 (1.00–1.77)	1.73 (1.27–2.35)
Brunei	2442	1.94 (1.48–2.55)	1.32 (1.00–1.74)
Cayman Island	1148	1.32 (0.92–1.90)	1.29 (0.90–1.86)
Cook Island	641	1.26 (0.76–2.06)	1.54 (0.97–2.45)
Costa Rica	2579	1.35 (1.09–1.67)	1.36 (1.10–1.70)
Curacao	1971	1.37 (1.01–1.85)	1.41 (1.05–1.90)
Djibouti	1661	1.18 (0.87–1.59)	1.67 (1.25–2.24)
Ecuador	4978	1.19 (0.90–1.56)	1.83 (1.38–2.43)
Egypt	2285	0.90 (0.68–1.19)	0.97 (0.74–1.26)
El Salvador	1802	1.04 (0.76–1.42)	1.19 (0.88–1.62)
Fiji	2813	1.58 (1.30–1.92)	1.47 (1.21–1.79)
French Polynesia	2667	1.18 (0.95–1.47)	1.26 (0.99–1.58)
Ghana	2316	1.60 (1.22–2.08)	1.77 (1.35–2.32)
Grenada	1341	1.71 (1.23–2.37)	1.61 (1.12–2.33)
Guyana	2245	1.01 (0.79–1.30)	1.33 (1.03–1.71)
Honduras	1646	1.12 (0.84–1.49)	1.61 (1.19–2.16)
India	7241	1.51 (1.32–1.74)	1.85 (1.62–2.10)
Indonesia	10,553	1.27 (1.11–1.46)	1.43 (1.25–1.63)
Iraq	1876	1.74 (1.32–2.30)	1.64 (1.24–2.17)
Jordan	2066	2.05 (1.50–2.82)	2.22 (1.61–3.05)
Kiribati	1520	1.45 (1.00–2.11)	1.71 (1.26–2.32)
Kuwait	2937	1.44 (1.15–1.80)	1.59 (1.27–1.97)
Laos	3546	1.34 (1.03–1.74)	1.06 (0.81–1.38)
Lebanon	2088	1.39 (1.04–1.86)	1.79 (1.29–2.49)
Libya	2015	1.45 (1.10–1.91)	2.00 (1.53–2.63)
Macedonia	1887	1.65 (1.20–2.28)	1.36 (1.01–1.83)
Malaysia	24,930	1.61 (1.47–1.76)	1.72 (1.57–1.88)
Mauritania	1839	1.48 (1.01–2.16)	2.04 (1.40–2.96)
Mongolia	5026	1.50 (1.30–1.73)	1.56 (1.34–1.80)
Morocco	2613	1.24 (0.96–1.61)	1.49 (1.17–1.91)
Mozambique	1242	1.58 (1.04–2.41)	1.00 (0.66–1.52)
Myanmar	2732	1.45 (1.13–1.85)	2.11 (1.66–2.68)
Namibia	3158	1.30 (1.05–1.61)	1.55 (1.24–1.93)
Nauru	460	1.10 (0.54–2.24)	1.47 (0.79–2.77)
Nepal	5948	1.64 (1.37–1.97)	1.83 (1.52–2.19)
Oman	2961	1.56 (1.23–1.99)	2.17 (1.66–2.85)
Pakistan	4926	0.85 (0.70–1.02)	0.93 (0.76–1.14)
Palestine	13,369	1.55 (1.37–1.75)	1.65 (1.45–1.87)
Peru	2809	1.61 (1.29–2.01)	1.84 (1.47–2.31)
Philippines, The	8201	1.39 (1.13–1.70)	1.43 (1.18–1.75)
Qatar	1559	2.34 (1.67–3.30)	2.52 (1.79–3.56)
Saint Kitts and Nevis	1625	1.28 (0.93–1.76)	1.46 (1.00–2.12)
Saint Lucia	1189	1.56 (1.10–2.19)	1.25 (0.87–1.82)
Saint Vincent and the Grenadines	1159	2.06 (1.45–2.95)	1.73 (1.15–2.60)
Samoa	1776	1.46 (1.03–2.06)	1.93 (1.37–2.71)
Seychelles	2284	1.29 (1.01–1.66)	1.37 (1.05–1.79)
Solomon Island	1231	1.60 (1.07–2.41)	1.32 (0.89–1.95)
Sri Lanka	2480	1.35 (1.04–1.75)	1.79 (1.34–2.41)
Sudan	1972	1.51 (1.05–2.18)	1.51 (1.04–2.19)
Suriname	1565	1.18 (0.91–1.53)	1.36 (1.04–1.76)
Syria	2983	1.50 (1.15–1.97)	1.57 (1.21–2.04)
Tanzania	3455	1.81 (1.51–2.18)	1.88 (1.57–2.25)
Thailand	5432	1.47 (1.14–1.89)	1.56 (1.22–1.98)
Timor-Leste	2796	2.03 (1.35–3.07)	1.70 (1.24–2.32)
Tonga	2103	1.56 (1.18–2.07)	2.45 (1.88–3.20)
Trinidad and Tobago	2535	1.17 (0.91–1.51)	1.83 (1.41–2.38)
Tunisia	2728	1.49 (1.22–1.82)	1.42 (1.16–1.74)
Tuvalu	834	1.09 (0.55–2.14)	1.50 (0.85–2.63)
United Arab Emirates, The	2394	1.45 (1.14–1.83)	1.55 (1.19–2.01)
Uruguay	3303	0.97 (0.77–1.21)	1.26 (0.99–1.59)
Vanuatu	1058	2.15 (1.19–3.87)	1.48 (0.85–2.58)
Vietnam	3012	1.38 (1.09–1.75)	1.44 (1.13–1.82)
Wallis and Futuna	912	1.17 (0.78–1.76)	1.54 (1.00–2.39)
Yemen	2089	2.12 (1.52–2.94)	1.88 (1.37–2.59)

^1^ Adjusted for age, gender, BMI, and food insecurity.

## References

[1] Poitras V.J., Gray C.E., Borghese M.M., Carson V., Chaput J.-P., Janssen I., Katzmarzyk P.T., Pate R.R., Connor Gorber S., Kho M.E. (2016). Systematic review of the relationships between objectively measured physical activity and health indicators in school-aged children and youth. Appl. Physiol. Nutr. Metab..

[2] World Health Organization (2010). Global Recommendations on Physical Activity for Health.

[3] Guthold R., Stevens G.A., Riley L.M., Bull F.C. (2020). Global trends in insufficient physical activity among adolescents: A pooled analysis of 298 population-based surveys with 16 million participants. Lancet Child Adolesc. Health.

[4] Uddin R., Lee E., Khan S., Tremblay M., Khan A. (2020). Clustering of lifestyle risk factors for non-communicable diseases in 304,779 adolescents from 89 countries: A global perspective. Prev. Med..

[5] Ng M., Fleming T., Robinson M., Thomson B., Graetz N., Margono C., Mullany E.C., Biryukov S., Abbafati C., Abera S.F. (2014). Global, regional, and national prevalence of overweight and obesity in children and adults during 1980–2013: A systematic analysis for the Global Burden of Disease Study 2013. Lancet.

[6] Gustafson S.L., Rhodes R.E. (2006). Parental correlates of physical activity in children and early adolescents. Sports Med..

[7] Yao C.A., Rhodes R.E. (2015). Parental correlates in child and adolescent physical activity: A meta-analysis. Int. J. Behav. Nutr. Phys. Act..

[8] Haidar A., Ranjit N., Archer N., Hoelscher D.M. (2019). Parental and peer social support is associated with healthier physical activity behaviors in adolescents: A cross-sectional analysis of Texas School Physical Activity and Nutrition (TX SPAN) data. BMC Public Health.

[9] Pyper E., Harrington D., Manson H. (2016). The impact of different types of parental support behaviours on child physical activity, healthy eating, and screen time: A cross-sectional study. BMC Public Health.

[10] Reimers A.K., Schmidt S.C., Demetriou Y., Marzi I., Woll A. (2019). Parental and peer support and modelling in relation to domain-specific physical activity participation in boys and girls from Germany. PLoS ONE.

[11] Neshteruk C.D., Nezami B.T., Nino-Tapias G., Davison K.K., Ward D.S. (2017). The influence of fathers on children’s physical activity: A review of the literature from 2009 to 2015. Prev. Med..

[12] Henriksen P., Ingholt L., Rasmussen M., Holstein B. (2016). Physical activity among adolescents: The role of various kinds of parental support. Scand. J. Med. Sci. Sports.

[13] Solomon-Moore E., Toumpakari Z., Sebire S.J., Thompson J.L., Lawlor D.A., Jago R. (2018). Roles of mothers and fathers in supporting child physical activity: A cross-sectional mixed-methods study. BMJ Open.

[14] Kirby J., Levin K.A., Inchley J. (2011). Parental and peer influences on physical activity among Scottish adolescents: A longitudinal study. J. Phys. Act. Health.

[15] Salvy S.-J., Roemmich J.N., Bowker J.C., Romero N.D., Stadler P.J., Epstein L.H. (2009). Effect of peers and friends on youth physical activity and motivation to be physically active. J. Pediatr. Psychol..

[16] Lau E., Faulkner G., Qian W., Leatherdale S. (2016). Longitudinal associations of parental and peer influences with physical activity during adolescence: Findings from the COMPASS study. Health Promot. Chronic. Dis. Prev. Can..

[17] Pengpid S., Peltzer K. (2016). Parental involvement, health behaviours and mental health among school-going adolescents in six Asian countries. ASR Chiang Mai Univ. J. Soc. Sci. Humanit..

[18] Pengpid S., Peltzer K. (2018). Parental involvement, health behaviour and mental health among school-going adolescents in seven Pacific Island countries. J. Hum. Behav. Soc. Environ..

[19] Kitayama S., Uchida Y. (2005). Interdependent agency: An alternative system for action. Cultural and Social Behavior: The Ontario Symposium.

[20] Mora T., Gil J. (2013). Peer effects in adolescent BMI: Evidence from Spain. Health Econ..

[21] World Health Organization Global School-Based Student Health Survey (GSHS). https://www.who.int/ncds/surveillance/gshs/en/.

[22] US Centers for Disease Control and Prevention Global School-Based Student Health Survey (GSHS). https://www.cdc.gov/gshs/.

[23] Ashdown-Franks G., Vancampfort D., Firth J., Veronese N., Jackson S.E., Smith L., Stubbs B., Koyanagi A. (2019). Leisure-time sedentary behavior and obesity among 116,762 adolescents aged 12–15 years from 41 low- and middle-income countries. Obesity.

[24] Uddin R., Khan A. (2019). Sedentary behaviour is associated with overweight and obesity among adolescents: Evidence from a population-based study. Acta Paediatr..

[25] Keane E., Kelly C., Molcho M., Nic Gabhainn S. (2017). Physical activity, screen time and the risk of subjective health complaints in school-aged children. Prev. Med..

[26] Carver A. (2010). Are children and adolescents less active if parents restrict their physical activity and active transport due to perceived risk?. Soc. Sci. Med..

[27] Woldeamanuel M. (2016). Younger teens’ mode choice for school trips: Do parents’ attitudes toward safety and traffic conditions along the school route matter?. Int. J. Sustain. Transp..

[28] Mandic S., Hopkins D., García Bengoechea E., Flaherty C., Coppell K., Moore A., Williams J., Spence J. (2020). Differences in Parental Perceptions of Walking and Cycling to High School According to Distance. Transp. Res. Part F Traffic Psychol. Behav..

[29] Chung S.J., Ersig A.L., McCarthy A.M. (2017). The Influence of Peers on Diet and Exercise Among Adolescents: A Systematic Review. J. Pediatr. Nurs..

[30] Finnerty T., Reeves S., Dabinett J., Jeanes Y.M., Vögele C. (2010). Effects of peer influence on dietary intake and physical activity in schoolchildren. Public Health Nutr..

[31] Yan J.H., McCullagh P. (2004). Cultural Influence on Youth’s Motivation of Participation in Physical Activity. J. Sport Behav..

[32] Chen H., Sun H., Dai J. (2017). Peer support and adolescents’ physical activity: The mediating roles of self-efficacy and enjoyment. J. Pediatri. Psychol..

[33] Lijuan W., Jiancui S., Suzhe Z. (2017). Parental influence on the physical activity of Chinese children: Do gender differences occur?. Eur. Phys. Educ. Rev..

[34] Liu Y., Zhang Y., Chen S., Zhang J., Guo Z., Chen P. (2017). Associations between parental support for physical activity and moderate-to-vigorous physical activity among Chinese school children: A cross-sectional study. J. Sport Health Sci..

[35] Khalaf A., Ekblom Ö., Kowalski J., Berggren V., Westergren A., Al-Hazzaa H. (2013). Female university students’ physical activity levels and associated factors—A cross-sectional study in southwestern Saudi Arabia. Int. J. Environ. Res. Public Health.

